# Seroprevalence of Viral Hepatitis B and C among Blood Donors in the Northern Region of Riyadh Province, Saudi Arabia

**DOI:** 10.3390/healthcare9080934

**Published:** 2021-07-24

**Authors:** Saeed Mohammed Alqahtani, Suliman A. Alsagaby, Shabir Ahmad Mir, Mohammed Alaidarous, Abdulaziz Bin Dukhyil, Bader Alshehri, Saeed Banawas, Wael Alturaiki, Naif Khalaf Alharbi, Taif Anwar Azad, Waleed Al Abdulmonem

**Affiliations:** 1 Department of Medical Laboratory Sciences, College of Applied Medical Sciences, Majmaah University, Al Majmaah 11952, Saudi Arabia; saeed4462009@hotmail.com (S.M.A.); s.alsaqaby@mu.edu.sa (S.A.A.); m.alaidarous@mu.edu.sa (M.A.); a.dukhyil@mu.edu.sa (A.B.D.); b.alshehri@mu.edu.sa (B.A.); s.banawas@mu.edu.sa (S.B.); w.alturaiki@mu.edu.sa (W.A.); 2 Health and Basic Sciences Research Center, Majmaah University, Al Majmaah 11952, Saudi Arabia; 3 Department of Biomedical Sciences, Oregon State University, Corvallis, OR 97331, USA; 4 King Abdullah International Medical Research Center, Department of Infectious Disease Research, Riyadh 11451, Saudi Arabia; harbina2@ngha.med.sa; 5 King Saud Bin Abdulaziz University for Health Sciences, Riyadh 11451, Saudi Arabia; 6 Department of Ophthalmology, College of Medicine, King Saud University, Riyadh 11451, Saudi Arabia; taif.anwar@gmail.com; 7 Department of Pathology, College of Medicine, Qassim University, Buraidah 51452, Saudi Arabia; waleedmonem@qumed.edu.sa

**Keywords:** prevalence, hepatitis, HBV, HCV, blood donors, Saudi Arabia, blood transfusion

## Abstract

Background: Hepatitis B and C viral infections, which are the most common cause of liver infection worldwide, are major health issues around the globe. People with chronic hepatitis infections remain at risk of liver cirrhosis and hepatic carcinoma, while also being a risk to other diseases. These infections are highly contagious in nature, and the prevention of hepatitis B and C transmission during blood transfusion is a major challenge for healthcare workers. Although epidemiological characteristics of hepatitis B and C infections in blood donors in Saudi Arabia have been previously investigated in multiple studies, due to targeted cohorts and the vast geographical distribution of Saudi Arabia, there are a lot of missing data points, which necessitates further investigations. Aim of the study: This study aimed to determine the prevalence of hepatitis B and hepatitis C viral infections among blood donors in the northern region of Riyadh, Saudi Arabia. Methods: To determine the given objectives, a retrospective study was performed which included data gathered from serological as well as nucleic acid test (NAT) screening of blood donors. Clinical data of 3733 blood donors were collected for a period of 2 years (from January 2019 to December 2020) at the blood bank of King Khalid General Hospital and the associated blood banks and donation camps in the region. Statistical analysis of the clinical data was performed using SPSS. Results: The blood samples of 3733 donors were analyzed to determine the seroprevalence of hepatitis B and C among the blood donors in the northern region of Riyadh, Saudi Arabia. Among the total of 3733 blood donors, 3645 (97.65%) were men and 88 (2.36%) were women. Most of the donors were younger than 27 years of age (*n* = 1494). The most frequent blood group in our study was O-positive (*n* = 1534), and the least frequent was AB-negative (*n* = 29). After statistically analyzing the clinical data, we observed that 7 (0.19%), 203 (5.44%) and 260 (6.96%) donor blood samples were positive for the HBV serological markers HBsAgs, HBsAbs and HBcAbs, respectively, and 12 (0.32%) blood samples reacted positively to anti-HCV antibodies. Moreover, 10 (0.27%) and 1 (0.027%) samples were NAT-HBV positive and NAT-HCV positive, respectively. Conclusion: In the current study, low prevalence rates of HBV and HCV were observed in the blood donors. Statistical correlations indicated that both serological tests and NATs are highly effective in screening potential blood donors for HBV and HCV, which, in turn, prevents potential transfusion-transmitted hepatitis.

## 1. Introduction

Blood donation is the process of drawing blood from a healthy volunteer and collecting and storing it for transfusion into a patient with a matched blood group. Blood transfusion plays an important role in saving patient lives and stabilizing their medical conditions, but transfusion-transmitted diseases, via blood-borne pathogens, remain a medical concern. The most notable transfusion-related risks worldwide include HIV-AIDS and hepatitis. Hepatitis is a serious liver disease caused by a group of viruses. It is characterized by inflammation of the liver with a presence of inflammatory cells which are triggered by the infection and can lead to severe complication such as fibrosis or cirrhosis, which might be fatal [1]. The most common cause of hepatitis infections is a group of five viruses, namely, hepatitis virus A, B, C, D, and E. However, the major health concerns are primarily posed by the hepatitis B virus (HBV) and hepatitis C virus (HCV) due to their higher rates of infection, multiple modes of transmission, and initial stages of asymptomatic presence which delays detection, rendering the disease chronic in nature [2].

One-third of the world’s population (more than two billion people) are infected with HBV, which therefore poses a major health challenge; however, excluding immunocompromised individuals, 95% of adult hepatitis B patients recover with minimal or no clinical care [3]. In contrast, the recovery rate in children is below 10%. HBV could eventually result into liver cirrhosis and liver carcinoma [4]. Around 90% of HBV cases (either recovered or chronic) develop specific immunity against the condition [5]. On the other hand, about 170 million people are infected with HCV worldwide, and HCV is 10 times more infectious and transmissible as compared to HIV [6]. HCV is similar to HBV in terms of infectivity of the liver, but it could replicate in peripheral mononuclear cells of lymphoid tissue and bone marrow [7,8].

Hepatitis B and C viruses can be transmitted through both horizontal and vertical (perinatal) routes. Their infection occurs through contact with infected blood, blood products, unprotected sex with an infected person, or shared needles; additionally, an infected mother can transmit HBV to her baby before, during, or after childbirth [2]. Preventing these infections primarily relies on proper and timely vaccination as well as on the serological screening of blood donors using hepatitis B surface antigen (HBsAg) assays for HBV and anti-HCV antibody assays for HCV, respectively. In some countries, anti-hepatitis B core antigen antibody (HBcAb) assays are also being employed to detect chronic HBV carriers with a low viral load in blood without detectable HBsAg. Nucleic acid amplification testing (NAT) for HBV and HCV has also successfully been introduced to screen donors in many developed countries, including Saudi Arabia, over the past several years [9]. Approximately 2.3 billion people of the global population are infected with one or more hepatitis viruses, resulting in about 1.4 million deaths, 90% of which are caused by hepatitis B and C viruses [10,11,12]. Therefore, chronic hepatitis infections have resulted in an increased mortality rate and pose a serious burden on the healthcare systems of various countries, including Saudi Arabia. A substantial number of studies have been conducted in Saudi Arabia to study the prevalence of HBV and HCV in blood donors; however, limited research has been conducted on HBV and HCV prevalence rates in blood donors in urban settings of the country.

The overall reported prevalence rates of HBV and HCV in Saudi Arabia are approximately 3.2% and 1.2%, respectively [13], according to studies on various target groups including military personnel, healthcare staff, premarital screening, hemodialysis patients, pregnant women, and blood donors across various geographical distributions, as well as socioeconomic backgrounds [14]. However, due to the healthcare awareness program and vaccination drives and/or campaigns in recent decades, the prevalence of viral hepatitis has significantly decreased in the Saudi population. Nevertheless, there are cases of chronic viral infections and occult HBV and HCV in the blood of apparently healthy individuals, which poses a serious concern for blood transfusions [15,16].

For recognizing the risk of viral hepatitis infection burdens on blood transfusion and to evaluate the prevalence of hepatitis viral infections in blood donors, multiple studies have been conducted at different blood banks and hospitals from different regions of Saudi Arabia [17,18,19,20,21,22,23,24,25,26]. In addition, in 2018, our team reported the seroprevalence of some important transfusion transmitted infections (TTIs) in blood donors in Al Majmaah city, where the TTI prevalence was found to be comparatively low [23]. 

Here, we present a concentrated study on HBV and HCV infections and report the prevalence of their serological and molecular biomarkers among blood donors in the northern region of Riyadh province, Saudi Arabia. We also evaluated the efficacy of testing methods employed for the HBV and HCV screening of blood donors and correlated HBV and HCV infections with the presented socio-demographic variables, particularly the age and blood group of the donors.

## 2. Materials and Methods

### 2.1. Study Site and Population

The study was carried out in the Blood Bank Laboratory of King Khalid General Hospital (KKGH), located in Al Majmaah city, northern Riyadh province, Saudi Arabia. The trained staff screened the blood donors following the standard guidelines issued by the Saudi Food and Drug Authority (SFDA) [27] and Saudi Ministry of Health [28]. The selected donors underwent physical and clinical examination and also completed a donor questionnaire through which their complete history was acquired. The socio-demographic information presented in this study was obtained from these donor-filled questionnaires. The samples and data were collected for a period of two years, from January 2019 to December 2020. 

### 2.2. Sample Processing

Blood samples were obtained from all eligible donors, and the sample from each donor was equally divided into two parts. One part was transferred to an ethylene diamine tetra acetic acid (EDTA) containing a vial and stored at 2–4 °C after gentle swirling and subsequently used for plasma preparation within 24 h. The plasma was prepared by centrifuging the blood sample at 3000 RPM for 5–10 min at 4 °C. The resulting supernatant (plasma) was transferred into another vial using a pipette and stored at 2–4 °C and analyzed within 24 h.

The other part of blood sample was transferred to a plain tube (without any anticoagulant) and left undisturbed for 20–30 min at room temperature. The sample was then centrifuged at 3000 RPM for 5–10 min in a refrigerated centrifuge at 4 °C. The yellowish supernatant (serum) was separated, transferred to a vial using a pipette, stored at 2–4 °C, and analyzed within 24 h. 

### 2.3. Screening of Serum/Plasma Samples

The serum samples were screened for HBV and HCV serological markers using the Architect i1000SR immunoassay analyzer (Abbott, Abbott Park, IL, USA), following the instructions of the manufacturer. An ARCHITECT HBsAg Qualitative II Reagent kit, ARCHITECT Anti-HBs reagent kit, ARCHITECT Anti-HBc II Reagent kit and ARCHITECT Anti-HCV Reagent kit were used to perform these assays. All these analytical kits for the serological markers were procured from Abbott (Abbott Park, IL, USA). A reading equal to or greater than 1.00 was considered reactive, whereas less than 1.00 was considered non-reactive.

For screening with a nucleic acid amplification test (NAT), HBV and HCV nucleic acid quantification and detection were carried out for all plasma samples using CobasTaqScreenMPX Test, version 2.0 (Roche molecular diagnostics, Basel, Switzerland). The MPX assay was run on a Cobas s 201 system (Roche molecular diagnostics, Basel, Switzerland), as per the manufacturer’s instructions.

### 2.4. Statistical Analysis

Statistical analysis was conducted using SPSS version 21.0 and MedCalc version 19.8, with the help of an expert biostatistician. Descriptive analysis was performed to identify infection frequencies in donors using both datasets from the serological immunoassay and NAT screening. Statistical evaluations of various parameters in blood donor dataset were also performed to understand their mean values and general distributions in the study population.

Chi-squared cross-tabulation tests and kappa inter-rater correlation tests were used to assess these comparisons. *p*-values of ≤0.05 were considered statistically significant, with 95% confidence intervals for all correlation analyses. Any significant association between the prevalence of serological markers and NATs was explored without bias. 

### 2.5. Ethical Approval

Patient confidentiality and identity was safeguarded as per the research guidelines required by the hospital governing body. Ethical and institutional approval for the study was obtained from the King Fahad Medical City Institutional Review Board and associated Ethical Committees (IRB approval number: 20-787E).

## 3. Results

**Figure 1:**
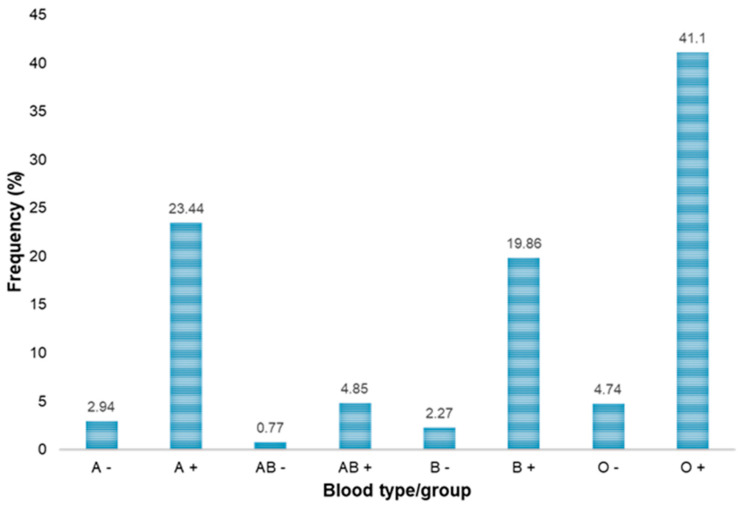
ABO/Rh blood group types of the studied blood donors.

In this observational retrospective study, we collected and analyzed the demographic and clinical data of 3733 blood donors in the northern Riyadh region, over a period of 2 years (from January 2019 to December 2020). Out of the 3733 blood donors, 3645 (97.65%) were men and 88 (2.35%) were women. The average age of the donors included in this study was 31.95 ± 10.31, with 86.36% of the donors being Saudi citizens, whereas 13.64% donors were from other nationalities. Donor demographics and their frequencies are summarized in Table 1. The most frequent blood group in the donor population was O-positive (41.1%), followed by A-positive (23.44%) as presented in Figure 1. The donors were categorized into different age groups, and most of the donors (40%) were in the age group of 18–27 years (Table 1). The blood units collected from the donors were subjected to screening for various transfusion-transmitted diseases to prevent or reduce the chances of infections in blood transfusion. Various markers were used for different infectious viral diseases; in case of hepatitis B and C, which are covered in this study, samples were tested using serological markers. All samples were also screened using multiplex nucleic acid amplification tests (NATs). Of all donor blood samples, 7 (0.19%) tested serologically positive for HBsAg, whereas 260 (6.96%) and 203 (5.44%) were positive for anti-HBc and anti-HBs antibody tests, respectively (Table 2). In addition, 12 (0.32%) of the donor samples were reactive for anti-HCV antibodies. Moreover, the nucleic acid amplification test (NAT)-based screening of the blood samples revealed that 10 (0.27%) and 1 (0.027%) donors tested positive for NAT-HBV and NAT-HCV, respectively (Table 3).

The vital signs for the donors were also recorded before the blood donation. Almost all the donors were healthy and did not present with any discomfort or abnormal physiology; hence, the vital signs were in the normal range. Hemoglobin levels for all blood donors were also observed to be within the normal range required for blood donation. The blood pressure values of the donors included in our study were also within the acceptable normal limits, ranging between 60 and 90 mmHg for diastolic pressure and 100 and 140 mmHg for systolic pressure, with few outliers of around 150 mmHg systolic pressure, which could be related to anxiety before blood donation, as has been suggested before [29].

The serologically positive and NAT-positive donors were categorized into different age groups to calculate and identify the age group with higher prevalence of the disease markers. As shown in Table 4, the highest prevalence of most of the serological markers and NAT was observed in the 48–57age group, followed by the 38–47 age group. Furthermore, categorizing the serologically positive and NAT-positive donors on the basis of their ABO/Rh blood group, the HBcAb, HBsAb, HBsAg, anti-HCV, NAT-HBV and NAT-HCV markers were most prevalent in AB− (13.79), AB− (10.34), B− (1.18%), B− (1.18%), B− (1.18%) and O+ (0.06%) blood groups, respectively (Table 5).

Statistical analysis was conducted to determine correlations between serological tests and NATs using the kappa inter-rater agreement test for determining associations between functional variables, as reported previously [30]. Cross-tabulation chi-squared tests were also performed to check significance between any two markers. Our analysis revealed that there were weak or no correlations between NAT-HBV and the serological markers of HBV (Table 6). The correlation was also assessed between NAT-HCV and the anti-HCV antibody test, which again showed no correlation, as indicated by the low kappa value of 0.15 between them (Table 7). In all correlation analyses, the positive findings of NATs were in agreement with serological markers but not vice versa; this supports the fact that both screening parameters are effective in combination to prevent the transfusion-transmitted hepatitis B and C viruses.

## 4. Discussion

Blood transfusion is a vital medical practice, and screening blood for various infectious agents is one of its main components—and concerns. Adequate screening of blood before transfusion is of utmost importance, because incidences of transfusion-transmitted infections (TTIs) are becoming the primary cause of mortalities and morbidities in various populations worldwide [31]. Several viruses have been identified as being transmitted via blood transfusion, including HBV, HCV, HTLV, and HIV [32]. These TTIs place significant burdens on healthcare providers around the world, including in Saudi Arabia. Therefore, TTI screening is an essential step in decreasing blood- and blood product transfusion-related biohazards.

In Saudi Arabia, most of the blood bank laboratories, including that of KKGH, use all of the established serological tests and NATs for screening blood donors. Several studies have reported on the prevalence of various transfusion-transmitted viral diseases in different regions and cities of Saudi Arabia. However, keeping in view the regional differences in disease prevalence and to support the periodic update of the prevalence data in the country, the current study was conducted to estimate the prevalence of HBV and HCV among blood donors of the northern region of Riyadh province. Here, we have retrospectively investigated the incidence of the serological markers and NATs, used for the diagnosis of HBV and HCV, among blood donors. A total of 3733 blood donors were screened for HBV and HCV over a period of 2 years. In addition to donating blood, all the donors included in this study provided socio-demographic information. Out of 3733 donors, 3645 (97.65%) were men and 88 (2.35%) were women. This gender difference is due to the fact that female blood donors are uncommon in Saudi Arabia as compared to other countries. The majority of the donors were 18 to 37 years old, whereas the fewest donations came from older age groups, i.e., 57 years and above. Additionally, the most common blood type was O+ (41.1%), and the least common blood type was AB− (0.77%). A similar distribution of blood groups has been reported by other studies conducted in Saudi Arabia [23,33]. The results of serological assays on serum samples from the donors showed that 7 (0.19%), 260 (6.96%), 203 (5.44%) and 12 (0.32%) donors reacted to HBsAg, HBcAb, HBsAb and anti-HCV, respectively (Table 2).

The seroprevalence of HBsAg among the blood donors included in this study was found to be 0.19%. Previously, various seroprevalence rates for HBsAg have been reported among blood donors in different countries, with 9.8%, 4.1% and 0.087% prevalence rates observed in Nigeria, Ethiopia and Serbia, respectively [34,35,36]. Therefore, as evident from our results, the prevalence rate of HBsAg among blood donors in the current study was low. In Saudi Arabia, regional variations in HBV prevalence are well established in the literature. For example, 3%, 5.4%, 1.5% and 0.3% prevalence rates of HBsAg have been reported among blood donors in the northwest region of Saudi Arabia, the city of Tabuk [37], southwest Saudi Arabia [38], the central region of Saudi Arabia [39] and the Al-Baha region of Saudi Arabia [25], respectively. The HBV prevalence observed in the present study (0.19%) and in our earlier study (0.33%) [23], both of which were performed in the north of the Riyadh region, revealed a notable decrease in HBsAg prevalence among the blood donors over time, between 2018 and 2019–2020. El Beltagy et al. proposed risk factors such as increased age, being married, lower educational level, specific occupations such as a blue-collar worker and the military, family history of HBV infection, and lack of immunization, to be associated with the prevalence of HBV infection [37]. The low prevalence of HBV reported in our study could be attributed to the inclusion of an HBV vaccine and the expanded program of immunization (EPI) in 1998. A study conducted on blood donors from the Al-Baha region showed that the inclusion of an HBV vaccine in the EPI was associated with at least a seven-fold decline in the prevalence of HBV [25]. Mass vaccination against HBV and the increasing public awareness about HBV infection are the key factors playing important roles in decreasing the prevalence of HBsAg in Saudi Arabia [26,40]. The seroprevalence of HBcAb/anti-HBc, another serological marker of HBV, has also reportedly declined in Saudi Arabia, from 15.32% to 9.15% between the years 1998 and 2001 [17]. The downward trend in the prevalence of anti-HBc appears to have continued ever since, and in the present study, we observed a 6.96% prevalence rate of anti-HBc antibody among the blood donors, which is lower compared to that reported in our previous study (9.81%) in 2018, for the same region.

The prevalence of HCV infection in the present study was 0.32%, which is slightly lower than that reported in our previous study [23]. Prevalence assessed from Saudi blood donor screening centers indicates HCV infection rates of 0.4–1.1% [37,41,42]. Two studies from Riyadh reported a 1.1% prevalence rate of HCV in 2003 [43] and 0.4% prevalence rate of HCV in 2004 [39] among blood donors, which indicates a notable decline in the prevalence of HCV. The 0.32% prevalence rate of HCV among blood donors from the northern Riyadh region in the present study suggests the persistence of low-level HCV among blood donors in Riyadh over time. The inclusion of an HCV vaccine in the EPI contributed to the drop (4.3-fold decrease) in the HCV prevalence [25]. However, future studies should assess the prevalence in at-risk groups such as drug addicts, people who are sexually active with multiple partners—if possible to identify—and prisoners.

This study represents a multi-national donor population in Saudi Arabia; therefore, it is limited by the difficulty to assess the prevalence with regard to changing expatriate populations living in Saudi Arabia. The tested samples only covered a duration of two years, which made it difficult to draw clinically significant conclusions for the representative donor population of Saudi Arabia living in the target region. Large-scale studies are recommended to validate the findings of this study. The detection of HBsAg is the basis for an excellent HBV donor screening test; however, HBsAg tests have certain limitations, including a lack of detection during the acute window period (WP) and mutations in the major hydrophilic region of the surface antigen [44,45]. The development and implementation of HBV DNA tests in the late 1990s, which were subsequently adapted to blood donation screening in the form of multiplex (HIV/HCV/HBV) NAT, contributed to the reduction in missed cases resulting from the acute HBV WP [46,47]. After proper correlation analysis of the retrospective data presented in this study, we observed that there were weak or no correlations between NAT and the serological tests for HBV and HCV, indicating that NAT as well as the serological test are important and more effective when combined for screening for HBV and HCV. The current study should support the global effort of eradicating HBV and HCV chronic diseases, rather than eradicating the virus itself, by the year 2030, as supported by the WHO and many key players worldwide [48,49,50]. This effort includes better surveillance, detection, documentation, and prophylactic and therapeutic measures [50,51].

## 5. Conclusions

In this study, we observed low prevalence rates of both HBV and HCV among blood donors in the northern region of Riyadh. The prevalence of HBV and HCV appears to have declined over the years since the introduction of the HBV vaccination program and combined serology and NAT testing in Saudi Arabia. It is evident from the study that combinations of serological as well as NAT tests are most effective for screening the donors and reducing the risk of transfusion-related hepatitis infections. NAT should be used for blanket screening for all blood donors in order to reach the goal of zero transmission risk. More studies should be conducted to assess the awareness among blood donors as well as the general population about the risk of infection. More studies should be aimed at certain population where the risk of HBV and HCV can be higher than the healthy donors, in order to develop a better estimate of the prevalence rates of the viruses.

## Figures and Tables

**Table 1:** Demographic characteristics of blood donors who donated blood at KKGH in Al Majmaah city or at the associated blood donation camps in other cities of the region, from January 2019 to December 2020.

Demographic Characteristics		Frequency, *n* (%)
**Nationality**	Saudi nationals	3224 (86.36)
	Other nationalities	509 (13.64)
	Total	3733 (100)
**Gender**	Male	3645 (97.65)
	Female	88 (2.35)
	Total	3733 (100)
**City**	Majmaah	2636 (70.6)
	Artawiah	229 (6.1)
	Riyadh	248 (6.64)
	Tumair	156 (4.17)
	Al-ghat	133 (3.6)
	Zulfi	83 (2.22)
	Other cities	248 (6.64)
	Total	3733 (100)
**Age**	18-27	1494 (40)
	28-37	1193 (32)
	38-47	719 (19.3)
	48-57	265 (7.1)
	58-65	57 (1.5)
	>65	5 (0.1)
	Total	3733 (100)

**Table 2:** The prevalence of HBV and HCV serological markers in the blood samples of donors.

Serological Marker/Screening Test	Test Result	Number of Donor Samples	Total Percentage
HBsAg	Negative	3726	99.81%
	Positive	7 (7 Male; 0 Female)	0.19%
HBsAb	Negative	3530	94.56%
	Positive	203 (198 Male; 5 Female)	5.44%
HBcAb	Negative	3473	93.04%
	Positive	260 (255 Male; 5 Female)	6.96%
Anti-HCV	Negative	3721	99.68%
	Positive	12 (12 Male; 0 Female)	0.32%

**Table 3:** Prevalence of a positive nucleic acid amplification test (NAT) for HBV and HBC in the blood samples of donors.

NAT Screening Tests	Test Result	Number of Donor Samples	Total Percentage
NAT-HBV	Negative	3723	99.73
	Positive	10 (9 Male; 1 Female)	0.27
NAT-HCV	Negative	3732	99.97
	Positive	1 Male only	0.027

**Table 4:** Distribution of serologically or NAT-positive donors among different age groups.

Age Groups (in Years)	Blood Donors	HBsAb-Positive, *n* (%)	HBsAg-Positive, *n* (%)	HBcAb-Positive, *n* (%)	Anti-HCV Positive, *n* (%)	NAT-HBV Positive, *n* (%)	NAT-HCV Positive, *n* (%)
**18–27**	1494	28 (1.87)	2 (0.13)	53 (3.55)	1 (0.07)	1 (0.07)	1 (0.07)
**28–37**	1193	56 (4.69)	2 (0.17)	73 (6.12)	3 (0.25)	3 (0.25)	0 (0.00)
**38–47**	719	66 (9.18)	2 (0.28)	75 (10.43)	4 (0.56)	3 (0.42)	0 (0.00)
**48–57**	265	37 (13.96)	1 (0.38)	44 (16.60)	4 (1.51)	2 (0.75)	0 (0.00)
**58–65**	57	16 (28.07)	0 (0.00)	15 (26.32)	0 (0.00)	1 (1.17)	0 (0.00)
**>65**	5	0 (0.00)	0 (0.00)	0 (0.00)	0 (0.00)	0 (0.00)	0 (0.00)
**Total**	3733	203 (5.44)	7 (0.19)	260 (6.96)	12 (0.32)	10 (0.27)	1 (0.03)

**Table 5:** Distribution of serologically and NAT positive donors corresponding to their ABO/Rh blood groups.

ABO/Rh Blood Groups	Blood Donors	HBsAb-Positive, *n* (%)	HBsAg-Positive, *n* (%)	HBcAb-Positive, *n* (%)	Anti-HCV Positive, *n* (%)	NAT-HBV Positive, *n* (%)	NAT-HCV Positive, *n* (%)
**A−**	110	3 (2.73)	0 (0.00)	3 (2.73)	0 (0.00)	0 (0.00)	0 (0.00)
**A+**	875	49 (5.60)	0 (0.00)	63 (7.20)	2 (0.23)	1 (0.11)	0 (0.00)
**B−**	85	1 (1.18)	1 (1.18)	5 (5.88)	1 (1.18)	1 (1.18)	0 (0.00)
**B+**	742	40 (5.39)	3 (0.40)	54 (7.28)	5 (0.67)	5 (0.67)	0 (0.00)
**AB−**	29	3 (10.34)	0 (0.00)	4 (13.79)	0 (0.00)	0 (0.00)	0 (0.00)
**AB+**	181	11 (6.08)	0 (0.00)	13 (7.18)	0 (0.00)	0 (0.00)	0 (0.00)
**O−**	177	10 (5.65)	0 (0.00)	16 (9.04)	0 (0.00)	0 (0.00)	0 (0.00)
**O+**	1534	86 (5.61)	3 (0.19)	102 (6.65)	4 (0.26)	3 (0.20)	1 (0.06)
**Total**	3733	203 (5.44)	7 (0.19)	260 (6.96)	12 (0.32)	10 (0.27)	1 (0.027)

**Table 6:** Correlations between NAT-HBV and HBV serological markers.

Parameters		NAT-HBV					
		Number of Negative Units	Percentage	Number of Positive Units	Percentage	*p*-Value	Kappa Value
**HBsAg**	Negative	3720	99.65%	6	0.16%	<0.001	0.469
	Positive	3	0.08%	4	0.11%		
**HBcAbs**	Negative	3468	92.9%	5	0.13%	<0.001	0.32
	Positive	255	6.84%	5	0.13%		
**HBsAbs**	Negative	3528	94.5%	2	0.06%	<0.001	0.07
	Positive	195	5.23%	8	0.21%		

**Table 7:** Correlation between NAT-HCV and HCV serological markers.

Parameters		NAT-HCV					
		Number of Negative Units	Percentage	Number of Positive Units	Percentage	*p* Value	Kappa Value
**Anti-HCV**	Negative	3721	99.67%	0	0%	<0.001	0.15
	Positive	11	0.3%	1	0.03%		
